# Perioperative hyperbaric oxygen therapy: mechanisms, evidence, and clinical application

**DOI:** 10.4103/mgr.MEDGASRES-D-25-00318

**Published:** 2026-09-12

**Authors:** Takudzwa Gavaza, Nathanael Matei

**Affiliations:** Departments of Physiology and Pharmacology, Loma Linda University School of Medicine, Loma Linda, CA, USA

**Keywords:** angiogenesis, fibroblast proliferation, hyperbaric oxygen therapy, hyperoxia, inflammation modulation, ischemia-reperfusion injury, microvascular perfusion, stem cell mobilization, tissue oxygenation, wound healing

## Abstract

Hyperbaric oxygen therapy delivers 100% oxygen at supra-atmospheric pressures, elevating plasma oxygen content and tissue oxygenation above normoxic levels. Once limited to decompression illness, hyperbaric oxygen therapy is gaining recognition as a perioperative adjunct with potential to mitigate complications through supra-physiologic tissue oxygenation. This review summarizes current evidence on the biological mechanisms, clinical applications, and considerations of perioperative hyperbaric oxygen therapy. Mechanistically, hyperbaric oxygen therapy has been shown to promote angiogenesis through redox-sensitive upregulation of hypoxia-inducible factor-1α and vascular endothelial growth factor, accelerate fibroblast proliferation and collagen synthesis, mobilize CD34-positive progenitor cells, and mitigate ischemia–reperfusion injury via antioxidant and anti-inflammatory pathways. Clinically, hyperbaric oxygen therapy has demonstrated improved graft uptake, flap salvage, and muscle recovery across trauma, reconstructive, and orthopedic contexts, and has shown promise in cardiac surgery preconditioning. The safety of hyperbaric oxygen therapy is favorable, with serious complications occurring in fewer than 0.5% of treatments. Despite accumulating evidence of its biological effect, widespread perioperative adoption remains constrained by cost, coverage limitations, and protocol heterogeneity. Higher-quality studies, including standardized dosing parameters and multicenter randomized trials, are needed to define optimal timing and patient selection. As health systems move toward precision perioperative optimization, hyperbaric oxygen appears to be gaining ground as a compelling, evidence-supported tool to improve surgical outcomes while aligning with the broader goals of restorative and regenerative medicine.

## Introduction

Hyperbaric oxygen therapy (HBOT) delivers 100% oxygen at supra-atmospheric pressures within a sealed chamber, substantially increasing plasma oxygen content and tissue oxygen tension.^1^ Originally developed for decompression illness, HBOT has evolved into a versatile therapeutic tool for hypoxic, ischemic, and infectious conditions.^1^,^2^

The perioperative period is divided into three phases: preoperative, intraoperative, and postoperative (**Figure 1**).^3^ For this review, preoperative HBOT refers to treatments with the last session ≤ 72 hours before surgery, whereas postoperative HBOT refers to treatments initiated ≤ 2 weeks after surgery. HBOT administered during surgery would be considered intraoperative; however, we found no reports of intraoperative HBOT.

**Figure 1:**
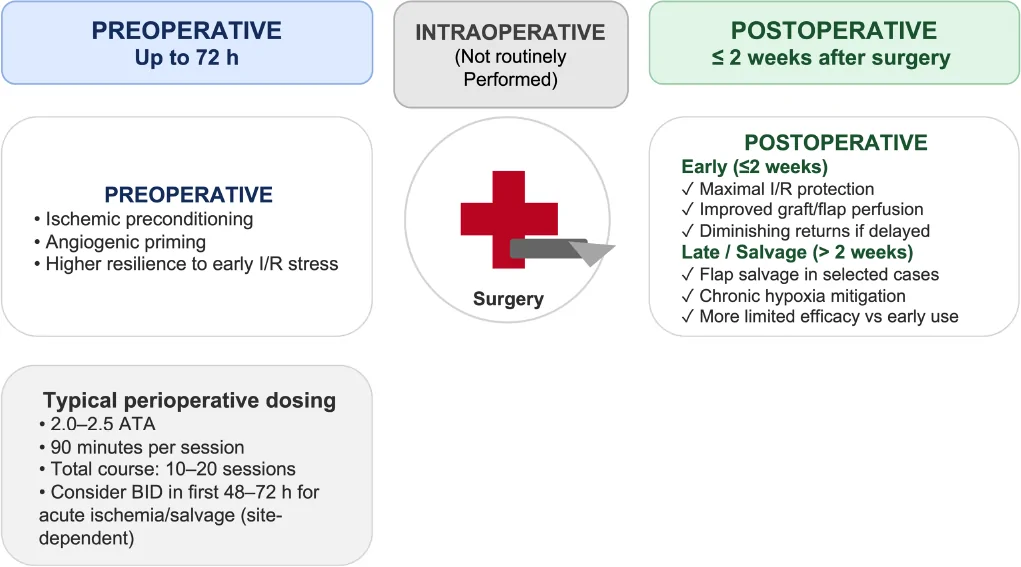
Timing and dose–response framework for perioperative hyperbaric oxygen therapy (HBOT). Initiation soon after surgery or at the first signs of flap/graft compromise is most likely to enhance I/R protection, support perfusion-dependent healing (e.g., graft uptake/flap viability), and temper inflammation. Benefit declines with delay, as established necrosis, infection, or microvascular failure becomes less reversible and more dependent on operative revision and source control. The figure separates late postoperative/salvage use (> 2 weeks) as a setting where HBOT may still help selected patients, especially when chronic hypoxia or radiation-related microvascular injury constrains healing, but with generally less predictable benefit than early perioperative initiation. Created with Microsoft PowerPoint for Mac (version 16.83). ATA: Atmospheres absolute; BID: twice daily; HBOT: hyperbaric oxygen therapy; I/R: ischemia–reperfusion.

While case series and early randomized trials have demonstrated promising outcomes across trauma, orthopedic, reconstructive, and cardiovascular interventions, the use of HBOT in perioperative protocols remains limited. This review discusses the physiological mechanisms, clinical evidence, and limitations of perioperative HBOT. It highlights the mechanisms by which hyperoxia influences surgical recovery, the procedural standards for therapy delivery, and the timing factors to maximize efficacy.

## Methods

We performed a narrative literature review to synthesize clinical evidence on perioperative HBOT. MEDLINE, PubMed and, when available, Embase and the Cochrane Library, were searched from inception to December 31, 2025, and reference lists of relevant reviews and high-yield primary studies were hand-searched to identify additional articles. Search terms combined keywords and controlled vocabulary for HBOT and perioperative surgery (e.g., “hyperbaric oxygen,” “HBOT,” “perioperative,” “preoperative,” “intraoperative,” “postoperative,” “surgery,” “reconstruction,” “flap,” “graft,” “ischemia-reperfusion,” “radiation injury,” “orthopedic”), with database-specific adaptations. In addition to database searching, we used artificial intelligence (AI)-assisted discovery tools (ChatGPT in Deep Research mode and the browser version) to identify potentially relevant articles not found with keyword searches. All AI-suggested records were treated only as leads and were independently screened. Articles were included if the relevant study population met criteria for perioperative HBOT as defined in the introduction and the study reported at least one clinically relevant outcome. Articles were excluded if they were not published in English.

## Administration Routes of Hyperbaric Oxygen Therapy

HBOT is administered by exposing the patient to 100% oxygen at pressures exceeding atmospheric levels within a sealed chamber. Delivery methods vary depending on chamber configuration and oxygen interface. The three primary configurations include monoplace chambers, which are single-patient acrylic tubes pressurized entirely with oxygen; multiplace chambers, which accommodate several patients and are pressurized with air while individuals breathe 100% oxygen via a hood or mask; and portable or transportable chambers, which are used less frequently in critical care but can be used to deliver HBOT outside of traditional healthcare facilities.^3^,^4^

A HBOT session in clinical practice typically lasts 90 to 120 minutes, including compression, therapeutic dwell, and decompression phases. During compression, chamber pressure rises gradually over 10–15 minutes. The target therapeutic pressure, which depends on the indication, is typically between 2.0–3.0 absolute atmospheres (ATA; 1 ATA = 101 kPa).^5^ The dwell phase lasts 60–120 minutes, during which the patient breathes 100% oxygen, sometimes with short “air breaks” to reduce oxygen toxicity. Finally, decompression reduces pressure back to ambient over 10–15 minutes to prevent barotrauma.^5^,^6^ Some protocols allow two or even three treatments daily in acute scenarios (e.g., carbon monoxide poisoning, gas embolism). On the other hand, most chronic wound or graft protocols are limited to one session per day.^7^ The total number of sessions depends on the indication; for example, nonhealing wounds often require 20–60 sessions, while acute injuries like carbon monoxide poisoning may require fewer.

Interface systems vary by chamber and patient condition. In monoplace chambers, the environment itself contains 100% oxygen, whereas multiplace chambers deliver oxygen through transparent hoods, oronasal masks, or endotracheal tubes for mechanically ventilated patients.^4^ Critically ill or postoperative patients can receive HBOT while intubated, with pressure-compatible ventilator circuits and continuous monitoring of end-tidal carbon dioxide, airway pressures, and oxygen saturation.

Standardization is affected by variability among chamber manufacturers and facility protocols. To reduce procedural errors, the U.S. Food and Drug Administration recommends following the manufacturer’s instructions, providing staff training, and performing continuous chamber inspection.^8^

## Mechanism of Perioperative Hyperbaric Oxygen Therapy

HBOT exerts its therapeutic potential through a variety of biological mechanisms that enhance tissue oxygenation, modulate inflammation, promote angiogenesis, and accelerate wound remodeling (**Additional Table 1**).^9^,^10^,^11^,^12^,^13^,^14^,^15^,^16^,^17^,^18^,^19^,^20^,^21^,^22^,^23^,^24^,^25^,^26^,^27^,^28^,^29^,^30^ When there is an increase in partial pressure of oxygen in plasma under hyperbaric conditions, tissue oxygen tension is increased 10–20 times baseline and allows oxygen diffusion into hypoperfused zones independent of hemoglobin oxygen saturation.^5^ This hyperoxia not only restores oxygen-dependent metabolic functions but also triggers a complex cascade of signaling pathways that contribute to wound repair and cellular recovery (**Figure 2**).

**Additional Table 1:** Mechanism of HBOT

Study	Year	Evidence type	Model/population	HBOT exposure	Comparator	Mechanistic endpoints	Main findings	Clinical translation
Fedorko et al.^9^	2016	RCT (clinical outcome)	Diabetic patients with chronic foot ulcers (pre-amputation risk)	30 HBO *vs.* 26 sham (chamber with air), over 12 wk	Sham (room air in chamber)	Wound healing endpoints (amputation rate, closure)	No significant difference in rates of meeting criteria for major amputation between HBOT (11/49, ~22%) and sham (13/54, 24%). HBO did not significantly improve ulcer healing outcomes overall, though both groups received good wound care.	Adjunctive HBO did not markedly improve clinical healing in diabetic ulcers; raises caution that mechanistic benefits (like stem cell release) may not translate into clinical outcomes without proper patient selection
Capó et al.^10^	2023	Interventional (pre-post)	Patients with chronic diabetic wounds (HBOT + standard care)	2.2 ATA, 60 min, 20 sessions (over 4 wk)	No separate control (within-patient comparison)	Plasma oxidative stress markers (MDA, protein carbonyls); cytokines	Over 20 HBOT sessions, patients had significant reductions in plasma MDA and protein carbonyls (*P* < 0.05), and pro-inflammatory cytokines TNF-α, IL-1β declined, while growth factors (TGF-β, PDGF) increased *vs.* baseline. Wounds showed improved healing trajectories.	HBOT reduced systemic oxidative and inflammatory burden in chronic wounds; extrapolated to perioperative care, HBO may foster a more pro-healing systemic environment post-surgery
Steenebruggen et al.^11^	2023	Observational	Patients with inflammation (acute or chronic conditions)	2.4 ATA, 90 min single session	Before *vs.* after HBO	RBC deformability (ektacytometry)	A single HBO session significantly improved RBC deformability in patients (elongation index ↑, *P* < 0.05) regardless of inflammation status. This suggests improved capillary transit after HBO.	HBO can acutely enhance microcirculatory flow by making RBCs more flexible; perioperatively, this could translate to better tissue perfusion and oxygen delivery in inflamed or surgical tissues
Paprocki et al.^12^	2019	Observational	Patients with sudden sensorineural hearing loss (HBOT treated)	2.5 ATA, 60 min, 15 sessions (3 wk)	Healthy controls (baseline)	Serum oxidative stress index (TAC, MDA)	HBOT-treated hearing-loss patients showed significantly higher total antioxidant capacity and lower MDA after therapy compared to pretreatment (*P* < 0.05), reaching levels similar to healthy controls.	HBOT can normalize oxidative stress profiles in patients; by analogy, perioperative HBO might correct surgery-induced oxidative imbalances
Teksam et al.^13^	2019	Observational	Children with acute CO poisoning (treated with HBO)	2.5 ATA, 90 min ×1 (acute treatment)	Pre-HBOT *vs.* post-HBOT	Serum oxidative stress (TOS/TAC) ratios	Before HBOT, CO-poisoned children had very high oxidative stress index; one HBO session significantly improved antioxidant status (TAC ↑) and lowered oxidative stress ratio (*P* < 0.01). Clinical symptoms improved concurrently.	Even a single HBO can acutely restore redox balance after severe oxidative insult; supports use of HBO to quickly counteract perioperative oxidative spikes (e.g., ischemia-reperfusion events)
Sureda et al.^14^	2016	Observational (pre-post)	Patients with chronic non-healing wounds (during HBOT course)	2.4 ATA, 90 min, 20 sessions (4 wk)	No separate control (pre *vs.* post)	Erythrocyte & plasma antioxidant enzyme levels; MDA	After 20 HBOT sessions, RBC and plasma antioxidant enzymes (SOD, catalase) increased significantly (*P* < 0.05) and MDA levels dropped, indicating reduced oxidative damage. Improvements persisted 1 month after healing.	Repeated HBO can induce lasting antioxidant adaptations in surgical patients, potentially lowering postoperative oxidative injury
Merkow et al.^15^	2015	Observational (retrospective cohort)	346,000+ surgical patients (national sample, various surgeries)	N/A (outcomes database analysis)	N/A (no HBOT, general outcomes)	Causes of 30-day readmission (chart review)	SSI was the leading cause of unplanned readmissions (19% of cases), highlighting the impact of infections on recovery. This underscores a major target for interventions.	Although not about HBOT, it frames the problem: reducing SSIs (e.g., via adjuncts like HBOT improving oxygen-dependent bacterial killing) could significantly improve perioperative outcomes (fewer readmissions).
Gurdol et al.^16^	2010	Observational	Diabetic patients with foot ulcers (during HBOT course)	2.4 ATA, 90 min, 25–30 sessions (~5 weeks)	Baseline *vs.* during HBOT	Plasma procollagen I & III levels; NO and ADMA	HBOT was associated with increased plasma procollagen I/III levels during the treatment course (indicative of enhanced collagen synthesis); NO levels rose and ADMA (endogenous NOS inhibitor) fell, suggesting improved endothelial function.	Perioperative HBO might accelerate collagen deposition in wounds and improve vascular function via NO, potentially speeding healing in surgical patients
Sureda et al.^17^	2009	Observational (pre-post)	Healthy volunteer scuba divers (hyperoxic exposure model)	Single dive (~1.4 ATA for ~1 h underwater)	Pre-dive *vs.* post-dive	Antioxidant defense markers (RBC catalase, SOD, etc.); NO	A single hyperoxic dive significantly increased RBC antioxidant enzyme activities (↑ SOD, catalase) without signs of oxidative damage. NO levels were maintained, suggesting enhanced redox balance rather than oxidative stress.	Acute hyperoxia (comparable to mild HBO) can boost antioxidant defenses; analogous perioperative HBO might similarly strengthen patient’s oxidative stress resilience
Thom et al.^18^	2006	Observational	Healthy adults (volunteers)	2.0 ATA exposures (multiple sessions)	Baseline (pre-HBOT)	Circulating CD34^+^ progenitor cell count	Repeated HBOT sessions induced a dose-dependent rise in circulating CD34^+^ stem/progenitor cells (significant increase over first 20 sessions; higher mobilization at 2.5 *vs.* 2.0 ATA).	HBO mobilizes stem cells/angiogenic cells, potentially improving angiogenesis in surgical healing when pre/post-operative HBO is applied
Horino et al.^19^	2002	Observational	Cultured human dermal fibroblasts	Hypoxia *vs.* normoxia conditions	Normoxic culture	Collagen synthesis rate (procollagen processing)	Prolonged hypoxia increased post-transcriptional collagen production in fibroblasts (↑ collagen accumulation under low O_2_). Under normoxic/hyperoxic conditions, fibroblasts produced more collagen than in hypoxia.	Adequate oxygenation (via HBO) can normalize or enhance fibroblast collagen production in wounds, benefiting perioperative tissue repair
Dhamodharan et al.^20^	2019	Animal	Diabetic wound model in rodents (with tissue-specific Nrf2 manipulation)	Not specified (standard HBO protocol in experiment)	Control (normoxia/no HBO)	Wound healing outcomes; Nrf2 pathway activation	Nrf2 activation was required for HBO-enhanced wound healing in diabetic model (HBO increased wound closure and antioxidant responses, absent if Nrf2 disabled).	HBO’s oxidative stress signaling (Nrf2) aids healing; translation depends on similar pathway activation in humans
Yin et al.^21^	2015	Animal	Rat myocardial ischemia–reperfusion (heart attack) model	2.5 ATA, 2 h preconditioning before ischemia	No HBO (ischemia only)	Infarct size; HO-1 expression (Western blot)	Rats preconditioned with HBO had ~50% smaller infarcts after heart ischemia–reperfusion *vs.* non-HBO (*P* < 0.01), correlating with a 3-fold increase in HO-1 expression in myocardium. Pharmacologically inhibiting HO-1 negated HBO’s protective effect.	Pre-surgical HBO could induce cytoprotective enzymes (HO-1) that shield tissues from ischemia–reperfusion injury (e.g., during cardiac or transplant surgeries), improving organ salvage
Sun et al.^22^	2011	Animal	Rat model of focal cerebral ischemia (stroke)	100% O_2_ at 1 ATA (normobaric O_2_) for 4 h post-stroke	Room air (no O_2_ therapy)	Brain ATP levels; infarct size	Normobaric oxygen therapy improved brain ATP production and reduced infarct size by ~25% *vs.* untreated stroke rats. Suggests even supplemental oxygen (not hyperbaric) aided energy metabolism in ischemic brain tissue.	The principle that increasing oxygen availability (even without pressure) benefits ischemic tissue; in perioperative terms, HBO could further amplify this effect in compromised tissues (e.g., during flap or organ ischemia)
Liu et al.^23^	2008	Animal	Diabetic mouse model (wound healing)	2.4 ATA, 90 min daily, 10 d	Normoxia (diabetic controls)	Endothelial progenitor cell count; wound closure rate; VEGF levels	HBO significantly increased endothelial progenitor cell mobilization and homing to wound sites in diabetic mice, doubling wound closure rate *vs.* control. Wound VEGF levels were higher with HBO, indicating enhanced angiogenesis.	HBO’s regenerative effect via stem/progenitor cell recruitment in impaired healing; suggests perioperative HBO could similarly boost reparative cell delivery to surgical wounds, improving outcomes
Skyhar et al.^24^	1986	Animal	Dog model of acute compartment syndrome + hemorrhagic shock	2.0 ATA, 60 min (delayed 2 h post-injury) x 1	No HBO (injury untreated)	Muscle edema (wet weight ratio); muscle necrosis (Tc-99 uptake)	Delayed HBO treatment markedly reduced muscle edema (edema ratio ~1.01 *vs.* 1.15 in controls, *P* = 0.01) and necrosis (~1.05 *vs.* 1.96 in controls, *P* = 0.04) in dogs with compartment syndrome. HBO-treated muscle outcomes were close to normal controls.	Even delayed HBO can salvage ischemic muscle by limiting edema and cell death; supports use in trauma or surgical compartment syndromes to improve tissue viability
Schönrock et al.^25^	2023	In vitro	Human osteoblastic cells (bone-forming cells)	2.5 ATA exposure in cell chamber (single)	Normoxia (no HBO)	DNA double-strand breaks (γ-H2AX foci); cell viability	Hyperoxic exposure at 2.5 ATA for 90 min induced mild DNA strand breaks in osteoblasts in vitro (↑ γ-H2AX foci *vs.* normoxia), but cells recovered and viability wasn’t significantly affected, especially with antioxidant presence.	Perioperative HBO at therapeutic doses causes manageable oxidative DNA stress in bone cells, which might be mitigated by inherent cellular defenses and not impede bone healing (safety signal for HBO use in orthopedic patients)
Park and Park^26^	2018	In vitro (engineering)	Oxygen-generating hydrogel system (lab prototype)	Simulated HBO environment (hydrogel releasing O_2_)	No HBO (standard hydrogel)	Oxygen release profile; cell viability	Developed a hydrogel that locally generates oxygen (equivalent to ~hyperbaric levels) within wounds. Improved fibroblast viability and function in oxygenated hydrogel *vs.* control hydrogel.	Alternative to chamber HBO for local oxygen delivery; concept supports the importance of oxygenation in wound healing (perioperative localized use)
Modarressi et al.^27^	2010	In vitro	Human skin myofibroblasts (cell culture)	Normoxia *vs.* hypoxia exposures	Normoxia (21% O_2_)	Myofibroblast differentiation (α-SMA expression); collagen contraction	Hypoxia impaired myofibroblast differentiation (↓ α-SMA expression) and function (weaker collagen gel contraction). Normoxic conditions yielded more robust myofibroblast activity, implying adequate oxygen is needed for optimal wound contraction.	By alleviating hypoxia, HBO may maintain myofibroblast function in wounds, improving wound contraction and closure in postoperative healing
Park et al.^28^	1991	In vitro microbiology	Pseudomonas aeruginosa cultures + gentamicin antibiotic	100% O_2_ at 3.0 ATA during antibiotic exposure	Normoxia (ambient air)	Post-antibiotic effect duration	Exposure to hyperoxia (3 ATA O_2_) prolonged gentamicin’s post-antibiotic effect against *P. aeruginosa* by ~3 hours *vs.* normoxia, indicating enhanced bacterial growth suppression after drug removal. Likely due to oxygen-dependent bacterial damage.	Synergy between high O_2_ levels and antibiotics *in vitro*; Perioperative HBO might boost antibiotic effectiveness *in vivo*, potentially reducing surgical infection rates (especially for anaerobic/aerobic mixed infections).
De Wolde et al.^29^	2021	Systematic review	Mixed human and animal studies (various conditions)	N/A (review aggregated multiple protocols)	N/A	N/A	Found consistent pattern: HBOT causes a brief increase in pro-inflammatory signals (e.g. IL-6) followed by significant decreases in TNF-α, IL-1β, IL-6 with repeated sessions. Anti-inflammatory IL-10 responses were variable.	Perioperative HBO might temper surgical inflammation (reducing harmful prolonged inflammation) after an initial benign spike, aiding recovery
Brouwer et al.^30^	2020	Systematic review/meta-analysis	13 RCTs of HBOT for diabetic foot ulcers (546 patients total)	Various (mostly 2.0–2.5 ATA, 30–40 sessions)	Combined results *vs.* standard care	Wound healing, amputations (pooled outcomes)	Meta-analysis found HBOT did significantly increased complete ulcer healing rates but reduced risk of major amputation in ischemic diabetic foot ulcers. Benefits were more pronounced in patients with adequate perfusion.	Highest-level evidence suggesting HBO may improve healing in chronic ischemic wounds; supports a potential perioperative role in preventing amputations in vascular-compromised patients (with caveat of patient selection)

AMDA: Alzheimer’s disease-related; ATA: atmospheres absolute; ATP: adenosine triphosphate; CO: carbon monoxide; HBO: hyperbaric oxygen; HBOT: hyperbaric oxygen therapy; HO-1: heme oxygenase-1; IL: interleukin; MDA: malondialdehyde; NO: nitric oxide; Nrf2: nuclear factor erythroid 2-related factor 2; O_2_: oxygen; *P. aeruginosa*: *Pseudomonas aeruginosa*; PDGF: platelet-derived growth factor; RBC: red blood cell; RCT: randomized controlled trial; SOD: superoxide dismutase; SSI: surgical site infection; TAC: total antioxidant capacity; TGF: transforming growth factor; TNF: tumor necrosis factor; TOS: total oxidant status; VEGF: vascular endothelial growth factor; α-SMA: α-smooth muscle actin; γ-H2AX: phosphorylated H2AX histone.

**Figure 2:**
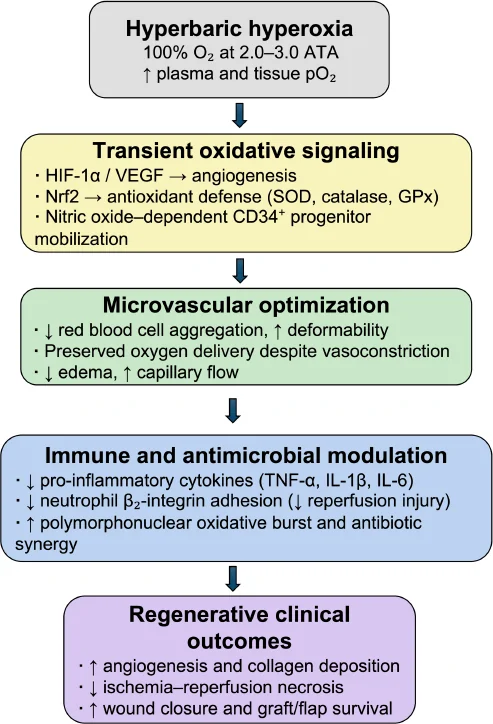
Mechanism of perioperative hyperbaric oxygen therapy. Exposure to 100% oxygen at supra-atmospheric pressures (2.0–3.0 ATA) increases plasma-dissolved and tissue pO2, enabling oxygen diffusion beyond hemoglobin-dependent perfusion. This hyperoxic exposure induces a controlled, transient oxidative signal that activates redox-sensitive pathways, including HIF-1α–mediated angiogenic signaling (e.g., VEGF), Nrf2-dependent antioxidant defenses (e.g., SOD, catalase, GPx), and nitric oxide-dependent mobilization of CD34^+^ progenitor cells. At the microcirculatory level, HBOT reduces erythrocyte aggregation and increases red blood cell deformability, improving capillary transit while oxygen-induced vasoconstriction limits tissue edema without compromising oxygen delivery. Concurrent immunologic and antimicrobial effects include attenuation of pro-inflammatory cytokines (e.g., TNF-α, IL-1β, IL-6), inhibition of neutrophil β_2_-integrin adhesion with reduced I/R injury, enhancement of polymorphonuclear leukocyte oxidative burst, and potentiation of select antimicrobial agents. Collectively, these integrated mechanisms promote angiogenesis and collagen deposition, reduce I/R–related necrosis, and improve wound closure and graft or flap survival in the perioperative setting. Created with Microsoft PowerPoint for Mac (version 16.83). ATA: Atmospheres absolute; GPx: glutathione peroxidase; HBOT: hyperbaric oxygen therapy; HIF-1α: hypoxia-inducible factor-1α; I/R: ischemia–reperfusion; IL: interleukin; Nrf2: nuclear factor erythroid 2–related factor 2; PMN: polymorphonuclear leukocyte; pO2: oxygen tension; RBC: red blood cell; SOD: superoxide dismutase; TNF-α: tumor necrosis factor-α; VEGF: vascular endothelial growth factor.

### Angiogenesis and vascular remodeling

HBOT promotes angiogenesis through the interaction between oxygen and hypoxia-inducible factor-1 (HIF-1). Although HIF-1α is upregulated under hypoxic conditions, repetitive hyperoxic exposure also induces transient oxidative stress that activates redox-sensitive transcription factors which increase HIF-1α expression.^31^ This upregulation increases downstream angiogenic mediators, such as vascular endothelial growth factor, fibroblast growth factor, platelet-derived growth factor, epidermal growth factor, and chemokine ligands 8 and 10, which coordinate endothelial cell migration and capillary sprouting.^20^ HBOT also increases bone marrow-derived stem cell mobilization via nitric oxide-dependent mechanisms, contributing to neovascularization and tissue regeneration,^18^ ultimately leading to improved blood flow and tissue healing. In summary, HBOT upregulates HIF-1α and increases angiogenic mediators, thereby stimulating angiogenesis and improving wound healing.

### Fibroblast proliferation and collagen synthesis

Fibroblasts, the key producers of extracellular matrix and collagen, exhibit oxygen-dependent enzymatic activity, particularly prolyl and lysyl hydroxylases, which are critical for collagen cross-linking.^19^,^32^ In a study involving 18 diabetic patients with foot ulcers, HBOT was found to be associated with increased fibroblast proliferation and upregulated collagen synthesis during the proliferative phase of wound healing.^16^ This study suggested that HBOT increased plasma levels of procollagen types I and III, which in turn were associated with improved wound healing. This increase in collagen deposition is most pronounced early after injury, underscoring the importance of initiating HBOT promptly.^33^ Experimental evidence indicates that oxygen tension influences collagen deposition.

### Reduction of oxidative stress

Oxidative stress is often exacerbated by hyperoxia in the form of excess reactive oxygen species. In the perioperative period, oxidative stress has been associated with worsened ischemia-reperfusion injury as well as cardiac injury, pulmonary toxicity and neurocognitive sequelae.^34^ HBOT acts as a controlled oxidative stimulus that has been associated with an induced adaptive antioxidant defense.^20^,^29^ HBOT has been shown to increase antioxidant levels, suggesting an adaptive response to oxidative stress.^14^,^17^ Other clinical studies also showed increased levels of the canonical antioxidant enzymes (superoxide dismutase, catalase, and glutathione peroxidase) after HBOT.^10^,^12^,^35^ In contrast, other studies reported no change or even decreases in antioxidant measures.^13^,^29^ Overall, these findings suggest that HBOT may precondition tissues to oxidative stress, thereby reducing susceptibility to ischemia-reperfusion injury, an effect that is particularly relevant in the perioperative setting.

### Modulation of inflammation

Inflammation is a critical component of wound healing by limiting infection and initiating tissue repair after injury.^36^ Clinically, HBOT is associated with an initial brief proinflammatory signal, likely due to its generation of transient oxidative stress.^29^ However, this early phase is quickly followed by an anti-inflammatory effect. This downregulation is reflected in decreased pro-inflammatory cytokines, such as tumor necrosis factor-α, and interleukin (IL)-1β and IL-6, though effects on anti-inflammatory mediators like IL-10 remain variable.^25^,^29^ HBOT has also been associated with inhibition of neutrophil β2-integrin adhesion, which may reduce leukocyte sequestration and endothelial damage in reperfusion zones.^5^ Together, this pattern of transient oxidative signaling followed by attenuation of maladaptive inflammation provides a plausible mechanistic basis for HBOT’s benefit in perioperative wound healing.

### Enhanced oxygen delivery and mitochondrial function

During HBOT, the patient breathes 100% oxygen at pressures of 2.0–3.0 ATA, increasing the partial pressure of inspired oxygen from ~160 mmHg to greater than 1400–2200 mmHg. This hyperoxia raises the arterial plasma partial pressure of oxygen to 1200–2000 mmHg, increasing dissolved plasma oxygen content from ~0.3 mL/dL to 4–6 mL/dL.^5^,^37^ Exposure to hyperoxia also reduces erythrocyte aggregation and increases red blood cell deformability, collectively improving capillary transit and oxygen diffusion.^11^,^38^ The rise in plasma-dissolved oxygen allows tissue oxygenation to be sustained despite oxygen-induced vasoconstriction, which decreases capillary hydrostatic pressure and limits edema formation.^39^,^40^ This increase in oxygenation also enhances mitochondrial respiration efficiency, supporting ATP production and cellular metabolism even in hypoperfused regions.^22^ Improved microcirculatory flow and reduced tissue edema are advantageous for recovery in postoperative wounds, crush injuries, and compartment syndromes.^24^,^39^,^40^,^41^ Overall, HBOT generates hyperoxia in tissues, which results in protective vasoconstriction, reducing edema while preserving oxygen-dependent cellular respiration.

### Stem cell recruitment and tissue regeneration

HBOT stimulates the mobilization of cluster of differentiation (CD) 34-positive progenitor cells from bone marrow into peripheral blood via a nitric oxide–dependent mechanism and activates the stromal cell-derived factor 1/C-X-C chemokine receptor type 4 axis, which is implicated in progenitor cell homing. In both human studies and *in vivo* diabetic wound models, there is augmented mobilization and recruitment of these stem cells to skin wounds.^18^,^23^ Repetitive sessions produce a dose-dependent rise in circulating CD34-positive cells, the greatest increase occurring during the first 20 sessions and higher mobilization at 2.5 *vs*. 2.0 ATA.^18^ Beyond cell trafficking, HBOT upregulates angiogenic and cytoprotective programs, including increased vascular endothelial growth factor *in vivo* and induction of heme oxygenase-1 through the phosphoinositide 3-kinase/protein kinase B/nuclear factor erythroid 2-related factor 2 pathway, thereby triggering an angiogenic/cytoprotective transcriptional response in human endothelial cells.^21^,^42^ One study found that HBOT enhances osteogenesis-angiogenesis coupling and bone repair in a tibial-defect mouse model.^43^ Also, a complementary human study confirms the progenitor-cell mobilization effect and its dose-dependent relationship with oxygen exposure.^44^ Collectively, these findings support HBOT as an active biologic stimulus that amplifies endogenous repair pathways.

### Inhibition of bacterial growth and biofilm disruption

Surgical site infection is among the most common preventable postoperative complications and remains a major driver of morbidity and delayed recovery.^15^,^45^ HBOT exerts both bacteriostatic and bactericidal effects by elevating tissue oxygen tension to levels exceeding the survival thresholds of anaerobic and facultative pathogens, disrupting metabolic enzyme systems, and inhibiting toxin synthesis.^46^ Increased oxygen availability enhances polymorphonuclear leukocyte oxidative killing, restoring phagocytic efficiency in hypoxic or ischemic tissue where the respiratory burst is normally impaired.^5^ As well as improving intrinsic infection fighting, a classic study showed that treatment with oxygen (including HBO) augments aminoglycosides against *Pseudomonas aeruginosa*, demonstrating enhanced post-antibiotic effect under 100% O_2_ and 3 ATA.^28^ Additionally, adjunctive HBOT has been reported to boost ciprofloxacin efficacy against biofilms, supporting synergy with select agents.^47^ Together, these mechanisms provide a rationale for perioperative HBOT as an adjunct to reduce infection risk and improve wound viability in hypoxic, contaminated, or high-risk surgical fields.

## Clinical Applications of Perioperative Hyperbaric Oxygen Therapy

The clinical translation of HBOT’s biological effects has been investigated for a growing number of perioperative indications. The therapy has increasingly been studied as a preoperative intervention to optimize surgical outcomes, minimize complications, and enhance recovery. A 2020 systematic review of 13 randomized controlled trials focused on perioperative HBOT found that most (10 of 13 trials totaling 546 total patients) trials showed positive effects of HBOT, while 2 trials showed no benefit and 1 showed a negative effect (**Figure 3**).^48^ Additional randomized trials also support potential benefit in selected contexts (**Additional Table 2**).^49^,^50^,^51^,^52^,^53^ However, the broader perioperative evidence base remains less robust and is dominated by other designs, highlighted in **Additional Table 3**.^30^,^48^,^54^,^55^,^56^,^57^,^58^,^59^,^60^,^61^,^62^,^63^,^64^ Unfortunately, HBOT treatment applications remain limited due to the prevalence of retrospective study designs, small sample sizes, and inconsistent procedures for administering therapy.

**Figure 3:**
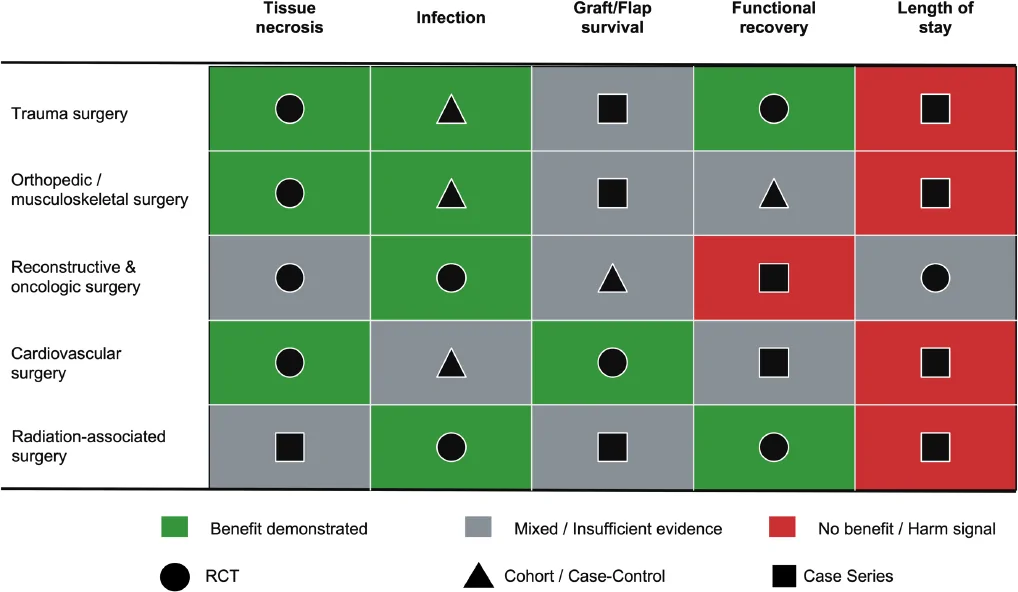
Evidence of perioperative HBOT by surgical domain. The most consistent clinical benefit of perioperative HBOT is observed when therapy is initiated early in ischemic or hypoperfused tissues, particularly for reducing necrosis and enhancing tissue viability, while also highlighting persistent limitations related to protocol heterogeneity, small sample sizes, and incomplete standardization across surgical specialties. Rows represent surgical contexts (trauma surgery; orthopedic and musculoskeletal surgery; reconstructive and oncologic surgery; radiation-injured or ischemic tissue reconstruction; cardiovascular surgery), and columns represent clinically relevant outcomes, including soft-tissue necrosis or ischemic loss, infection or wound complications, graft or flap survival, functional or muscle recovery, and length of stay or ICU metrics. Cell color denotes the overall direction of effect reported in the literature: green, benefit demonstrated; gray, mixed or insufficient evidence; red, no benefit or adverse signal. Created with Microsoft PowerPoint for Mac (version 16.83). HBOT: Hyperbaric oxygen therapy; ICU: intensive care unit; RCT: randomized controlled trial.

**Additional Table 2:** Randomized controlled trial evidence of HBOT

Study	Year	Setting	Population + procedure/condition	Randomization	Number	HBOT protocol	Timing relative to surgery/injury	Comparator	Primary endpoint	Key results	Follow-up	Risk of bias	Key limitation
Uniyal et al.^49^	2025	Single-center (India, trauma hospital)	Adults with post-traumatic large wounds requiring split-thickness skin graft closure	Randomized; open-label (no sham; investigators not blinded)	64 (HBOT *n* = 32; Control *n* = 32)	1.5 to 2 ATA; 90 min/session; daily sessions from POD 2	Immediate postoperative period (from graft placement until ~1 wk post-op)	Standard graft care (dressings, etc.) without HBOT	Graft uptake (%) on POD 4 and 7; donor site healing time	Graft takes significantly higher with HBOT: mean graft uptake on POD 4 was 92.4% *vs.* 88.1% (*P* = 0.036) and on POD 7 was 91.7% *vs.* 83.1% (*P* = 0.026). Donor site healing faster with HBOT (15.2 ± 0.9 days *vs.* 18.0 ± 2.5 d, *P* < 0.001). Control group had more graft-related complications (floating grafts in 2 patients *vs.* 0; flap edge necrosis in 4 *vs.* 0; 1 control patient died of sepsis).	15 d (until donor site healed)	Some concerns (open-label design, no blinded outcome assessment reported)	Single-center; short follow-up (2 wk) focusing on early wound outcomes; no long-term outcomes (e.g. graft functionality or cosmesis); possible imbalance in injury severity despite randomization (HBOT group had worse baseline radiation damage in other contexts).
Zhang et al. 50	2025	Single-center (China, tertiary hospital)	Adults with primary knee osteoarthritis undergoing total knee arthroplasty	Randomized; partial blinding (surgeons, data collectors, analysts blinded); no sham chamber (control received normobaric oxygen)	80 (HBOT *n* = 40; Control *n* = 40)	2.0 ATA; ~60 min/session (incl. 5 min air break); 1 session/d on POD 1 and POD 2 (total 2 sessions)	Immediate postoperative period (within 24–48 h after surgery)	Normobaric 100% O_2_ via nasal cannula (4 L/min for 48 h) + standard ERAS care	Serum muscle injury biomarkers (CK, LDH, AST/ALT, myoglobin) at 1, 3, 14 d post-op	Muscle damage markers significantly lower with HBOT on POD 3 (e.g. CK 3-day change: HBOT –27% *vs.* Control + 5%, *P* < 0.05); inflammatory markers (CRP, IL-6) also lower on POD 3 (CRP: –52% *vs.* –17%, *P* = 0.004). HBOT group had faster recovery in first 3 d: greater quadriceps strength, lower limb swelling, better ROM, and lower pain scores (VAS on POD 2–3, *P* < 0.05). No significant differences by 2 wk post-op; no increase in adverse events.	14 d (lab and functional outcomes); 6–12 wk (knee scores)	Some concerns (patients not blinded – no placebo chamber)	Single-center; short-term outcomes (≤ 2 wk) – unclear if early benefits translate to long-term functional improvement; primary endpoints are surrogate (biomarkers) rather than clinical outcomes; slightly underpowered sample.
Gušić et al. 51	2024	Single-center (Serbia, sports institute)	Elite youth soccer players after a 90-min competitive match (exercise-induced muscle fatigue)	Randomized; double-blind (both groups in chamber: HBOT *vs.* sham at 1.0 ATA)	20 (HBOT *n* = 10; Sham *n* = 10)	2.2 ATA; 60 min × 1 session (immediately post-match)	Post-exercise recovery (within 1–2 h after match)	Sham hyperbaric session at 1.0 ATA for 60 min (normobaric air)	Serum muscle injury markers (CK, LDH, AST, etc.) and performance tests (5–20 m sprint, vertical jumps) at 1 h and 12 h post-intervention; Hooper Index (fatigue/wellness score)	No significant differences between HBOT *vs.* control in any biochemical or performance metrics at any time point. Subjective recovery slightly improved with HBOT: Hooper Index 1 h post-treatment was lower (better) in HBOT group (8.6 ± 2.4 *vs.* 11.0 ± 3.2, *P* = 0.012).	12 h post-intervention	Low (robust randomization, sham control, double-blind)	Very small sample (*n* = 20) – potential Type II error; single session of HBOT only – may not reflect cumulative effects; limited to short-term recovery outcomes (no data on injury rates or long-term performance).
Millar et al. 52	2022	Multicenter (6 sites across 4 countries)	Adults with severe open tibial fractures requiring urgent orthopedic surgery (limb trauma)	Randomized; no patient blinding (no sham); outcome adjudication blinded	120 (HBOT *n* = 58; Control *n* = 59)	2.4-2.8 ATA; 80-100 min/session (with air breaks); 12 sessions over ~ 9 d	Early postoperative (HBOT started ≤48 h after injury)	Standard trauma care (surgery, antibiotics, etc.) with no HBOT	Composite of wound infection or necrosis by postoperative day 14	Composite infection/necrosis at 14 d was lower with HBOT (43% *vs.* 58%) but not significant (*P* = 0.12); tissue necrosis significantly reduced with HBOT (29% *vs.* 53%, OR 0.35, 95% CI 0.16–0.78; *P* = 0.01); fewer late complications (11% *vs.* 35%; *P* = 0.007) and improved 1–2 yr functional scores in HBOT group.	14 d (wound outcomes); 1–2 yr (functional outcomes)	Some concerns (open-label, no sham – potential performance bias)	Possibly underpowered for composite endpoint; no patient blinding could influence co-interventions; heterogeneity in injury severity; single-setting HBOT protocol (fixed 12-session regimen) – uncertain optimal dose.
Li et al.^53^	2011	Single-center (China, university hospital)	Adults undergoing elective coronary artery bypass grafting (on-pump or off-pump)	Randomized; single-blinded (outcome assessors blinded; no sham for control)	49 (On-pump: HBO *n* = 14 *vs.* Control *n* = 15; Off-pump: HBO *n* = 10 *vs.* Control *n* = 10)	2.5 ATA; ~60 min per session; 1 session/day for 5 d pre-op (5 total)	Preconditioning (daily HBOT for 5 ds immediately before surgery)	No HBOT (standard preoperative care only)	Perioperative markers of brain injury (S100B, neuron-specific enolase) and myocardial injury (troponin I); ICU length of stay; inotrope requirements	On-pump CABG: HBOT-preconditioned patients had significantly lower peak S100B, NSE, and troponin I levels than controls (all *P* < 0.05), indicating reduced cerebral and myocardial injury. HBOT group also had shorter ICU stays and less postoperative inotropic support. Off-pump CABG: no significant differences between HBOT and control in any outcomes.	In-hospital (ICU course and immediate postoperative period)	Some concerns (no patient blinding; small sample, especially in subgroups)	Small single-center trial; combined two surgical techniques – benefit observed only in on-pump subgroup, limiting generalizability; endpoints are surrogate biomarkers and short-term clinical measures (no long-term follow-up); no sham may allow bias in ICU care (though biomarker outcomes objective).

ALT: Alanine aminotransferase; AST: aspartate aminotransferase; ATA: atmospheres absolute; CABG: coronary artery bypass grafting; CI: confidence interval; CK: creatine kinase; CRP: C-reactive protein; HBOT: hyperbaric oxygen therapy; ICU: intensive care unit; IL-6: interleukin-6; LDH: lactate dehydrogenase; OR: odds ratio; POD: postoperative day; ROM: range of motion; VAS: Visual Analogue Scale.

**Additional Table 3:** Non-RCT evidence of HBOT

Study	Year	Evidence type	Population/model	Number	HBOT protocol	Comparator (if any)	Outcomes	Key finding	Key limitation
Leite et al.^54^	2024	Animal experiment	Rabbit model of anterior cruciate ligament reconstruction (auto-graft)	36 rabbits (~18 HBO *vs.* 18 control)	2.5 ATA; 120 min, daily for 5 days post-op	No HBOT (standard post-op care)	Graft healing on histology; biomechanical graft properties; tunnel widening on imaging	HBOT-treated rabbits showed better graft maturation and integration, stronger graft tensile properties, and significantly less tunnel widening *vs.* controls. HBOT group grafts had more organized collagen and higher failure load	Animal bone healing differs from human; no functional outcomes; small sample per group limits statistical power for some measures.
Oyaizu et al.^55^	2018	Animal experiment	Rat model of severe muscle contusion (traumatic calf injury)	Estimated 400 rats (randomly assigned HBO *vs.* control)	2.5 ATA, 120 min/d, starting 1 h post-injury, for 7 d	Sham treatment (normoxia)	Muscle edema (limb volume, wet weight); muscle function (contractile force); histologic regeneration; inflammatory response	HBOT reduced edema (lower limb swelling and muscle weight *vs.* controls) and improved muscle strength at 7 d. HBOT blunted the acute macrophage surge and then enhanced macrophage infiltration into injured muscle, increasing proliferating satellite cells and regenerating fibers. Enhanced IL-6/STAT3 signaling in early phase; overall dual effect of reducing inflammation and accelerating muscle repair.	Animal model – may not fully translate to humans; endpoints short-term (7 d); high HBOT dose (2.5 ATA, 2 h) not routinely used clinically.
Yagishita et al.^56^	2019	Comparative cohort study (non-randomized)	Human – 32 Japanese semi-professional rugby players with grade II medial collateral ligament (MCL) knee injury (non-operative management)	32 players (HBOT *n* = 16; No HBOT *n* = 16)	2.8 ATA, 60 min/session, 5 sessions within 10 d of injury	Standard sports injury rehab without HBOT	Pain (VAS during walking/jogging); time to return-to-play	Pain reduction (acute): VAS pain scores before *vs*. after a single HBOT session decreased (e.g. walking pain 37 → 32, *P* < 0.001). Recovery: HBOT group returned to play faster (31.4 ± 12.2 days *vs.* 42.1 ± 15.8 d, *P* < 0.05).	Allocation not random (possible selection of more motivated or less injured players for HBOT); unblinded outcome reporting; small sample; observational design cannot fully exclude placebo effect on pain reporting.
Vishwanath^57^	2011	Commentary (case series & review)	Human – composite discussion of free flap surgeries and adjunct HBOT	9 flap cases (with HBOT) in narrative	HBOT ~2.0 ATA, ~90 min, ~10–20 sessions (varied)	Literature/experience comparison (no direct control)	Free flap survival rates; author’s institutional outcomes with HBOT	HBOT’s role in free flap salvage is inconclusive – some anecdotal successes but overall flap success rates not clearly improved. Notes one series with ~60% success under HBOT, raising doubt if outcome differs from expected without HBOT.	No systematic data.
Scampa et al.^58^	2024	Retrospective comparative study	Human – post-mastectomy autologous microvascular breast reconstructions in irradiated fields	28 patients (HBOT *n* = 14; No HBOT *n* = 14)	HBOT 2.5 ATA, 90 min for ~20 sessions post-reconstruction	Matched cohort without HBOT	Flap perfusion (ICG angiography); wound-healing complications	HBOT was feasible and associated with qualitative perfusion improvement on imaging (better flap perfusion patterns). Fewer delayed wound-healing complications were observed in HBOT group (21% *vs.* 36%), but sample too small for statistical significance.	Very limited power; retrospective design; perfusion outcomes evaluated qualitatively; potential selection bias (patients with more severe RT damage may have been selected for HBOT).
Daniel et al.^59^	2025	Retrospective case series	Human – breast reconstruction patients with mastectomy skin flap ischemia (post-op)	23 patients (23 breasts)	HBOT 2.4 ATA, 90 min, mean 6 sessions; initiated as soon as ischemia noted (mean 1.4 d post-op)	No comparison group (all received HBOT)	Rate of flap necrosis requiring reoperation	Flap salvage in 79% of cases: 18 of 23 ischemic flaps healed without surgical re-intervention under HBOT (success defined as no major necrosis). 5 of 23 (21%) had partial necrosis needing surgical excision (2 cases progressed to reoperation despite HBOT).	No control group – cannot quantify HBOT benefit *vs.* natural healing; small sample; possible adjunct interventions uncontrolled; selection bias (only certain ischemic flaps offered HBOT).
Nasr et al.^60^	2023	Retrospective case series	Human – nipple-sparing mastectomy flaps with ischemia (threatened viability)	22 patients (25 breasts)	HBOT 2.0 ATA, 90 min; median 8 sessions; initiated on average 9 d post-op	No control (all received HBOT)	Flap salvage (avoidance of operative flap loss); HBOT side effects	Flap salvage achieved in 88% (22 of 25 breasts had successful recovery without further surgery). Only 3 of 25 (12%) ultimately required surgical reoperation (partial or full flap loss). One patient discontinued HBOT due to adverse effect (barotrauma). Later HBOT initiation (mean ~9 d) still yielded majority salvage.	No control group; potential selection bias of cases; varied timing of HBOT (some delays); small sample. High salvage rate might reflect inclusion of milder ischemia cases; unclear how many might have healed with standard care alone.
Spruijt et al.^61^	2021	Retrospective case series	Human – mastectomy skin flaps with ischemia post-breast reconstruction (immediate)	50 breasts (47 patients)	HBOT 2.0 ATA, 90 min, median 6 sessions (within 2 wk post-op)	No control (all received HBOT)	Need for surgical revision after HBOT; change in ischemic area size	No surgery required in 58% of cases after HBOT (i.e. 58% flap salvage rate). In remaining 42%, patients proceeded to surgical debridement or reoperation. Most cases showed reduction in ischemic area during HBOT (qualitative).	Lack of comparison – unclear what % might have avoided surgery with standard care alone; potential inclusion of milder ischemia cases; outcome assessment not blinded. Underlines variability in HBOT response (some flaps still failed).
Meier et al.^62^	2021	Retrospective case-control	Human – women undergoing delayed breast reconstruction after radiotherapy (RT) (flaps or implants)	45 patients (15 HBOT *vs.* 30 matched controls)	HBOT ~2.4 ATA, ~90 min for ~20 sessions (peri- and post-op)	Matched controls (no HBOT)	Postoperative complication rate (wound dehiscence, infection, flap loss)	Despite HBOT group having worse pre-reconstruction RT damage, their complication rates were similar to controls (no significant difference). HBOT patients achieved comparable healing outcomes to non-HBOT despite higher risk profile, suggesting a compensatory benefit.	Non-randomized and limited sample; matching was by RT damage extent and lifestyle factors but unmeasured confounders possible; outcomes were rare events, study underpowered to detect moderate differences.
Chang et al.^63^	2024	Retrospective cohort study	Human – adults with severe hand crush injuries requiring surgery (fasciotomy/repair)	72 patients (HBOT + standard care *n* = 36; standard care only *n* = 36)	HBOT ~2.0 ATA, ~90 min, ~18 sessions on average; started within 72 h post-op	Matched historical controls (no HBOT)	Wound healing time; hospital stay; number of additional surgeries; stratified by injury size and HBOT timing	HBOT group had shorter wound healing in smaller injuries (30 ± 13 *vs.* 41 ± 19 d, P = 0.03). Early HBOT (≤ 72 h) associated with shorter hospital stay (8 ± 6 *vs.* 16 ± 11 d, *P* = 0.04) and trend to faster healing (29 ± 18 *vs.* 41 ± 18 d, *P* = 0.08). Overall, HBOT patients required fewer reoperations (1.5 ± 0.8 *vs.* 2.4 ± 1.6, *P* = 0.06).	Retrospective design; HBOT group had larger wounds initially; possible referral bias (more severe cases received HBOT); lack of blinding and placebo.
Chen et al.^64^	2007	Retrospective analysis	Human – free flap transfers (various reconstructions) with postoperative vascular compromise	121 flaps (mixed types)	n/a (no HBOT in study; observational)	n/a	Time from surgery to first sign of flap ischemia; flap salvage success rate	When first signs of flap compromise occurred earlier, salvage success was significantly higher. Compromises presenting ≤ 24 h post-op had ~70% salvage *vs*. < 30% if detected > 24 h. Highlights critical window (most flap ischemia noted in first 72 h).	Not an HBOT study per se, but contextual evidence: underscores importance of early intervention (the rationale for early HBOT in compromised flaps). Data from 2007 techniques – may differ with modern monitoring.
Boet et al.^48^	2020	Systematic review of RCTs	Perioperative HBOT across diverse surgeries (13 RCTs included)	13 trials (*n* = 627 patients total)	Varied by included study (preventive or postoperative HBOT; doses 2.0–2.8 ATA)	Various controls (normobaric oxygen or standard care)	Aggregate patient outcomes (wound healing, complications, etc.)	10 of 13 trials found HBOT improved ≥ 1 outcome; overall positive trend for reduced complications. Heterogeneity precluded meta-analysis of all outcomes.	Mixed surgical contexts – results not generalizable to one domain; heterogeneity in HBOT dosing and endpoints limits definitive conclusions.
Brouwer et al.^30^	2020	Systematic review & meta-analysis	Human DFU with arterial insufficiency (peripheral vascular disease)	8 RCTs (n = 333) + 5 observational studies	HBOT 2.0–2.5 ATA, 60–120 min, ~20–40 sessions (adjunct to wound care)	Standard wound care (offloading, dressings ± revascularization)	DFU wound healing rate; major amputation rate	Pooled analysis showed no significant improvement in wound healing with HBOT *vs.* control. However, major amputation risk was reduced with HBOT (RR ~0.70, 95% CI ~0.54–0.90). Review notes patient selection (presence of critical ischemia) influenced outcomes, suggesting HBOT mainly helps avoid amputations in DFU with PAD.	Although focused on chronic wounds, cited to highlight heterogeneity in HBOT results. Applicability to peri-op setting is indirect. High heterogeneity in included studies; meta-analysis limited by small trials and variable endpoints.

ATA: Atmospheres absolute; CI: confidence interval; DFU: diabetic foot ulcers; HBO: hyperbaric oxygen; HBOT: hyperbaric oxygen therapy; ICG: indocyanine green; IL-6: interleukin-6; n/a: not applicable; PAD: peripheral arterial disease; RCT: randomized controlled trial; RR: relative risk; RT: radiation therapy; STAT3: signal transducer and activator of transcription 3; VAS: Visual Analogue Scale.

**Figure 4:**
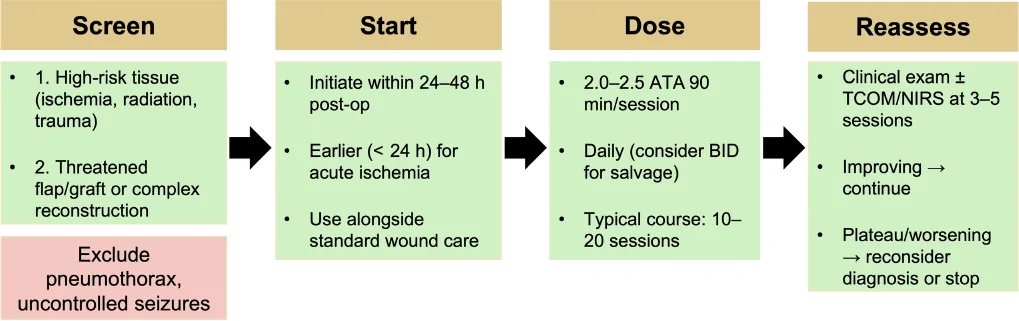
Synthesis-oriented perioperative HBOT framework. This synthesis-oriented framework integrates the reviewed mechanistic and clinical literature to illustrate a practical approach to considering perioperative HBOT across surgical contexts. It is intended to organize key decision points (e.g., identifying compromised tissue physiology, aligning timing with the perioperative course, and matching treatment intensity to clinical risk) and to highlight where evidence is strongest versus emerging. This framework is not a formal guideline, consensus statement, or prescriptive algorithm, and it should not replace clinical judgment or institution-specific protocols; rather, it is provided to help readers interpret heterogeneous study designs and translate the literature into a coherent, clinically relevant workflow. Created with Microsoft PowerPoint for Mac (version 16.83). ATA: Atmospheres absolute; HBOT: hyperbaric oxygen therapy; NIRS: near-infrared spectroscopy; TCOM: transcutaneous oximetry.

### Trauma surgery

Because traumatic injury creates a combined burden of hypoperfusion, inflammation, and oxidative stress, HBOT has been investigated as an early perioperative intervention in trauma surgery. In one multicenter randomized controlled trial of 120 adults with open tibial fractures, HBOT began within 48 hours of injury, and subsequent wound management surgery markedly reduced soft-tissue necrosis (29% *vs*. 53%) and lowered late complication rates such as delayed union and infection, leading to better long-term functional outcomes.^52^ Although the composite endpoint of infection plus necrosis at 14 days did not reach significance, the reduction in necrosis alone was substantial, emphasizing the value of HBOT therapy. While outcomes were not directly compared with late initiation vs. early initiation, early initiation of HBOT therapy after surgery appears to be more beneficial for healing.^63^ Based on these findings, it can be inferred that HBOT is most effective when impaired tissue viability and perfusion-limited wound healing drive the clinical pathology. What remains uncertain is the magnitude of benefit across trauma subtypes and modern limb-salvage pathways, as well as the optimal timing and total dose.

### Orthopedic and musculoskeletal surgery

In orthopedic and musculoskeletal surgery, adjunctive HBOT has shown promising results in reducing post-operative muscle injury and improving early functional recovery. In a recent randomized controlled trial of 80 patients undergoing primary total knee arthroplasty, HBOT, in addition to standard care, significantly reduced markers of muscle damage (creatine kinase, myoglobin, and lactate dehydrogenase) at day 3 compared with normobaric oxygen plus standard care.^50^ The study also found accelerated recovery of quadriceps strength and range of motion, reduced limb swelling, and was associated with lower inflammatory cytokine levels. Conversely, another study on HBOT’s effect on muscle healing, this time looking at minor injury during a 90-minute football game, found no significant improvement in recovery markers or performance outcomes after a single HBOT session.^51^ While there is evidence suggesting that HBOT therapy can improve healing of musculoskeletal injuries, this finding suggests that the difference may only be meaningful in more severe injuries, or more than one session may be required to achieve a noticeable benefit. Overall, HBOT is associated with improved biomarkers and physical exam findings in significant orthopedic injury.

Clinical data support the benefit of adjunctive HBOT in ligament injuries, with Yagishita and colleagues^56^ reporting that HBOT administered after grade 2 medial collateral ligament injury in rugby players was associated with faster symptom improvement and earlier return to play compared with standard care alone. While these data are not specific to perioperative HBOT, a currently recruiting clinical study addresses this gap by prospectively evaluating postoperative HBOT after anterior cruciate ligament reconstruction with standardized protocols and objective follow-up (NCT06493851). In summary, perioperative HBOT can reduce postoperative inflammation and support recovery when surgery or injury produces substantial soft-tissue insult accompanied by secondary hypoxia. What remains uncertain is whether these biologic and early functional effects translate into durable, clinically significant improvements under standardized protocols.

### Tumor and reconstructive surgery

Some of the most robust evidence for perioperative HBOT comes from surgical oncology and reconstructive surgery. Specifically, HBOT has been effective for breast reconstruction and skin grafts where tissue perfusion is a limiting factor. This distinction is especially relevant in patients undergoing reconstruction after radiotherapy, as radiotherapy can cause microvascular injury and fibrosis that can complicate surgical recovery.^65^,^66^ Despite this impaired healing environment, many patients still ultimately pursue reconstruction. Due to this need, HBOT has been a focus of research for reducing these surgical complications. In a retrospective case-control study of 45 women undergoing secondary breast reconstruction after radiotherapy, Meier et al.^62^ found that patients who received perioperative HBOT had significantly more severe baseline radiation damage yet achieved similar complication rates compared with lifestyle-matched and radiation-damage-matched control groups, suggesting a compensatory benefit of HBOT. A retrospective analysis by Scampa et al.^58^ in patients undergoing autologous microvascular breast reconstruction post-radiotherapy found that HBOT qualitatively improved perfusion and was associated with fewer delayed wound complications. While these studies suggest that HBOT may be a practical strategy to counteract radiotherapy-associated hypoxia and microvascular compromise during reconstruction, interpretation is limited by retrospective design and small sample sizes. Overall, HBOT has increasing clinical evidence for its beneficial role in breast reconstruction surgery, especially in non-ideal wound healing environments.

Similarly to breast reconstruction, survival of skin grafts is heavily dependent on early tissue perfusion, with the majority of flaps/grafts showing circulatory compromise within the first 72 hours.^64^ The evidence for the use of HBOT in this setting is mixed. One recent randomized controlled trial compared patients with traumatic wounds requiring split-thickness skin grafts who received either standard postoperative care alone or in combination with HBOT.^49^ The HBOT group demonstrated significantly greater graft uptake by post-operative day 4, with maintained graft uptake by day 7. The HBOT group also saw accelerated donor-site healing (15.16 *vs*. 17.97 days) compared to the control group. Another single-center retrospective study of 23 patients treated for mastectomy skin-flap ischemia found that early HBOT prevented major flap necrosis in the majority of cases (HBOT success rate ~79%).^59^

Nasr et al.^60^ reported a retrospective series of threatened nipple-sparing mastectomy flaps treated with HBOT, where flap compromise was due to ischemia, venous congestion, or partial-thickness necrosis. In this study, HBOT achieved flap salvage in 22 of 25 breasts (88%), with reoperation required in 3 breasts (12%), although therapy had to be stopped in one patient due to side effects of therapy. In contrast, other reports have found no clear benefit or lower recovery rates (e.g., 58%).^57^,^61^ These studies are difficult to compare directly because of likely selection bias from differing inclusion criteria and baseline severity, heterogeneity in HBOT protocols, and small sample sizes. There are also differences in HBOT timing, from immediate initiation to delayed initiation (mean ~9 days in the Nasr series). Taken together, these findings suggest that perioperative HBOT improves tissue viability in selected cases of threatened cutaneous perfusion. Still, larger, more robust controlled studies are needed to further clarify the magnitude of impact and cost-effectiveness of HBOT for this indication.

### Cardiovascular surgery

Recent literature in cardiac and vascular surgery frames HBOT in two main roles: as a preconditioning strategy before cardiac operations and as an adjuvant treatment for postoperative complications. HBOT’s mechanism of conditioning tissue to better withstand ischemia-reperfusion injury makes it an appealing adjuvant in coronary artery bypass grafting. A prospective, randomized, single-blinded study administered HBOT for 5 days before surgery to patients undergoing coronary artery bypass grafting (*n* = 24).^53^ Both the HBO and control groups were split into on-pump and off-pump groups, where “on-pump” refers to cardiopulmonary bypass, which has been associated with increased physiologic stress that can lead to postoperative complications via inflammatory response and ischemia-reperfusion injury.^67^,^68^ Patients pre-treated with HBOT in the on-pump group had lower perioperative levels of myocardial (troponin) and neuronal (S100B) injury markers and shorter length of stay and inotrope requirements in the intensive care unit compared with the off-pump control group.^53^ These protective effects were not observed in off-pump coronary artery bypass grafting, suggesting the benefit may be most relevant when cardiopulmonary bypass-associated ischemia-reperfusion and inflammatory stress are present. More recently, animal models have suggested the efficacy of preconditioning on improving acute kidney injury, a common complication of cardiac surgery.^38^,^69^ Overall, in high-risk cardiovascular procedures, there is promising evidence that HBOT may improve key metrics. What remains uncertain, however, is the consistency of benefit in contemporary practice, as well as the dose and timing required to affect primary postoperative outcomes, so the most appropriate next step is larger contemporary trials designed around patient-centered endpoints and standardized dosing.

Although many perioperative studies report improved perfusion-related outcomes, neutral or inconsistent findings likely reflect several recurring issues. First, there may be a diminishing return for increased oxygenation in settings where baseline tissue perfusion or oxygenation is already adequate. A key principle of therapeutic rationale for HBOT in compromised grafts is the augmentation of tissue oxygen tension. In uncompromised grafts, the effect of HBOT may not be sufficient to achieve a clinically significant difference. Second, null results may reflect an insufficient treatment dose. The literature on HBOT for complex wounds suggests front-loaded, repeated treatments and scaling based on clinical response.^70^,^71^ Some perioperative HBOT protocols are less intensive and may not deliver a comparable biologic stimulus for their intended indications. Third, especially in the setting of compromised grafts, expert guidance and reviews emphasize starting HBOT as soon as the compromise is identified.^59^,^72^ Delays in starting HBOT or in recognizing compromise may reduce its therapeutic efficacy. Notably, a systematic review of perioperative RCTs specifically notes substantial heterogeneity across surgical contexts, HBOT delivery, and outcomes, which precluded meta-analysis and firm practice recommendations.^48^ In summary, these methodological differences limit cross-study comparability and may mask their true therapeutic effects.

## Safety of Perioperative Hyperbaric Oxygen Therapy

Although HBOT has an excellent safety record, understanding potential adverse effects is essential for its safe clinical use and for contextualizing risk relative to routine perioperative morbidity. In most surgical pathways, clinicians routinely manage comparatively common complications such as perioperative hypoxemia, surgical-site infection, and tissue ischemia.^73^,^74^,^75^ HBOT’s adverse events are typically infrequent, predictable, and largely manageable, with serious events rarely reported.^76^ It is important to note that HBOT risk is not uniform and varies by treatment dose. A recent systematic review and meta-analysis of randomized trials found higher adverse-effect rates when chamber pressures exceeded 2.0 atmospheres absolute and when treatment courses extended beyond 10 sessions, consistent with cumulative exposure effects.^77^ Seizure risk was also reported to be directly proportional to treatment pressure. Pressure level, session duration, number of sessions, and patient comorbidities (most notably inability to equalize ear pressure), sinonasal disease, pulmonary pathology, and seizure predisposition, were other common risk factors for increased risk.^77^

The most common adverse event is middle ear barotrauma, resulting from pressure differentials across the tympanic membrane during compression and decompression. In a review of 5900 patients who were administered HBOT, 9.3% experienced ear barotrauma, but only 1.49% required treatment suspension.^78^ Other less frequent barotrauma events include sinus, pulmonary, and, rarely, dental or ocular involvement.^79^ Preventive measures include pre-treatment patient education on Valsalva techniques, use of nasal decongestants, and slow compression rates.

Patients with underlying pulmonary risk (e.g., obstructive disease, air-trapping, bullous disease) warrant careful screening and individualized protocols, especially when higher pressures or longer courses are planned. In the perioperative setting, risk assessment should be individualized and include a review of recent respiratory symptoms and available chest imaging to identify lung pathology. Treatment protocols can then be modified by using slower compression/decompression profiles, monitoring during ascent/descent, and multidisciplinary coordination when chest tubes or pleural drains are present, pausing treatment if adverse effects are observed.

Oxygen toxicity represents another key concern and is also dose- and host-dependent. Central nervous system oxygen toxicity may manifest as transient visual changes, nausea, or tonic-clonic seizures. In a large review, the seizure incidence was < 0.03% per session and fully reversible upon cessation.^6^ A 2025 multicenter analysis found a rate of approximately 1 per 10,000 treatments, without lasting neurologic sequelae.^80^ Pulmonary oxygen toxicity arises with prolonged exposure (> 6 hours/day) and is characterized by reversible cough, chest tightness, or mild vital capacity decline; however, deleterious outcomes are reduced via “air breaks.”^81^

Despite these theoretical risks, large-scale registry data confirm HBOT’s overall safety. Adverse event rates remain below 2%, with serious complications under 0.5%, most resolving with conservative management.^6^ Overall, perioperative HBOT has a low risk profile and is mainly associated with mild, manageable side effects. These risks increase with higher treatment pressures, longer courses, and certain comorbidities. HBOT does not appear to increase typical postoperative complications. The risk-benefit ratio remains strongly favorable, particularly in perioperative settings, where improved tissue oxygenation can improve surgical outcomes.

## Clinical Limitations of Perioperative Hyperbaric Oxygen Therapy

Widespread integration of HBOT into perioperative practice remains constrained by logistical, economic, and evidentiary barriers. Implementing HBOT programs requires not only specialized infrastructure but also a multidisciplinary workforce. Perhaps the most significant limiting factor in the perioperative use of HBOT is cost. Establishing or expanding an HBOT program requires substantial capital investment and ongoing compliance infrastructure, such as medical gas supply, ventilation, monitoring, and fire safety for oxygen-rich environments.

In the United States, Medicare coverage for HBOT is governed by National Coverage Determination 20.29, which limits reimbursement to a defined list of 15 covered indications and explicitly states that indications outside this list are noncovered.^41^ Under this determination, many perioperative use cases remain uncovered, shifting costs to hospitals and/or patients. Consequently, patients frequently incur out-of-pocket expenses, while hospitals bear substantial capital and compliance costs. Per-session costs have been estimated at approximately $595 in 2022, and although they have decreased overall between 2013 and 2022, the cumulative expense of typical perioperative protocols (often ≤ 20 treatments) can still be substantial.^82^

Operationally, HBOT also requires a multidisciplinary workforce involving credentialed physicians to supervise therapy, technologists trained in chamber operations, and nursing/respiratory staff capable of monitoring the patients. Undersea and Hyperbaric Medical Society guidance and accreditation-related materials emphasize these staffing expectations during chamber operations.^83^ While at high-volume centers, these requirements are manageable, they can be challenging for smaller hospitals to maintain. These capability gaps are particularly relevant for higher-acuity perioperative candidates. In a U.S. Survey of Hyperbaric Centers assessing readiness for emergencies or high-acuity cases, only 43 of 361 (11.9%) centers reported having the equipment and staff necessary to treat high-acuity patients, suggesting that many facilities function primarily as outpatient wound centers rather than perioperative-capable programs.^84^ Thus, even where chambers exist, delivering perioperative HBOT in a safe and timely manner may require institutional resources that are not universally available.

Importantly, the economic picture is indication-dependent. For example, claims-based analyses in radiation cystitis suggest that HBOT may reduce downstream resource use (fewer transfusions/procedures/hospitalizations) and may be associated with lower Medicare costs at 1 year in treated vs comparator cohorts, supporting the concept that HBOT can be economically favorable when it prevents expensive complications.^85^ However, comparable cost-effectiveness data for perioperative usage are sparse, and future perioperative trials should prospectively include economic endpoints to better align clinical evidence with coverage decision-making.

Another consideration is the emergence of other oxygen delivery modalities as credible alternatives to HBOT. Topical oxygen therapy involves delivering oxygen continuously at normobaric or low pressure directly to the wound surface. Similar to HBOT, topical oxygen therapy has strong evidence for use as an adjuvant in wound healing, especially in wounds with vascular compromise.^86^,^87^,^88^ Another alternative modality to HBOT is mild hyperbaric systems or mild HBOT. This treatment modality is similar to HBOT in concept; however, the pressures are lower, usually between 1.3–1.5 ATA.^39^ Mild HBOT appears to have a similar mechanism of action, but clinical evidence for effect and use cases remains limited, with most studies focusing on standard HBOT or normobaric modalities like topical oxygen therapy. These methods offer logistical advantages in outpatient or resource-limited settings but have limitations of their own. Despite these limitations, the potential for improved access, improved results, and lower costs associated with these modalities warrants further studies.^6^,^89^

## Design Limitations

Coverage remains narrow because perioperative HBOT studies are often heterogeneous in timing and dosing and frequently underpowered for clinically decisive endpoints, which limits guideline strength and makes payers reluctant to expand indications.^41^

A key limitation of the evidence base is poor comparability and external validity. Current perioperative HBOT studies differ widely in chamber type, pressure, session duration, total dose, and co-interventions, limiting meta-analytic synthesis and guideline strength for specific surgical specialties. Most HBOT protocols are cited between 2–3 ATA; however, there is significant variation between these pressures across studies and even within the same studies. Some studies have reported intragroup ranges of up to 0.5 ATA within the same study (**Additional Tables 2** and **3**). This heterogeneity makes it difficult to extrapolate a true minimum effective dose, in addition to limiting broader comparability.

Additionally, most available studies are small, single-center, or observational, and are often limited due to chamber availability and patient selection bias. Future work should consist of adequately powered, multicenter, protocol-harmonized trials that prioritize early initiation and standardized surgical endpoints (graft/flap salvage, necrosis rates, re-operation, time-to-ambulation, and perioperative quality of life). Additional gaps include optimal timing relative to surgery for each procedure (preconditioning *vs*. complication management) and establishing minimum effective pressures and session numbers.

Finally, the field lacks credible sham-controlled trials. Device studies are susceptible to expectation bias, and true sham-air controls are difficult to implement, especially in multiplace chambers. Of note, recent gastrointestinal HBOT trials in ulcerative colitis have successfully implemented blinded sham-air arms to mitigate bias and standardize co-therapies.^90^,^91^ Perioperative research can use this approach to strengthen internal validity and better isolate HBOT’s effects on surgical recovery. To date, there are multiple trials in varying stages of completion that aim to address this evidence gap, including breast reconstruction, flap necrosis, amputations, radiosurgery, and pancreaticoduodenectomy (NCT02575014, NCT03594344, NCT02714465, and NCT06811857). As the perioperative evidence base becomes more standardized and definitive, guidelines will evolve accordingly.

## Conclusion

Perioperative HBOT represents an evolving intersection between surgical innovation and translational physiology. Decades of evidence demonstrate that oxygen is not merely a passive substrate, but a molecule capable of regulating inflammation, angiogenesis, and cellular repair. With strategic administration around the time of surgery, HBOT has shown potential to enhance these processes at precisely the stage when tissues are most susceptible to hypoxia and reperfusion injury (**Figure 2)**. HBOT improves blood flow, supports tissue healing, and reduces infection risk, with relatively few safety concerns, making it a promising option in perioperative care.

Across surgical disciplines, the most consistent evidence demonstrates that the timing of HBOT is a key predictor of effectiveness. Early initiation appears to confer the most significant benefit, improving wound perfusion, graft adherence, and functional recovery. Interpretation of this literature is constrained by the narrative design of this review, which did not employ a preregistered systematic search strategy or uniform risk-of-bias adjudication across all included studies. Moreover, the underlying clinical evidence is heterogeneous in indications, HBOT dosing and timing, co-interventions, and outcome definitions, and is dominated in several domains by small trials and nonrandomized designs that remain vulnerable to confounding and publication bias. Nonetheless, the aggregate evidence supports HBOT as a potentially valuable adjunct when applied early and selectively in high-risk or ischemic perioperative settings.

The future of HBOT in perioperative care hinges on translating mechanistic promise into reproducible clinical outcomes. This will require addressing the gaps in current knowledge by improving protocol harmonization (including standardized pressure, duration, and frequency), integrating HBOT use into perioperative pathways (i.e., automatic consultation for high-risk reconstructions or ischemic wounds), and expanding policy-level coverage informed by multicenter trials. Continued efforts to improve the quality of evidence have the potential to shift the perception of HBOT from a niche therapy to a targeted intervention capable of enhancing the body’s intrinsic mechanisms of healing. As health systems move toward precision perioperative optimization, hyperbaric oxygen appears to be gaining ground as a compelling, evidence-supported tool to improve surgical outcomes while aligning with the broader goals of restorative and regenerative medicine.

**Facts**

1. Hyperbaric oxygen therapy (HBOT) increases plasma-dissolved oxygen and tissue oxygen tension, enabling diffusion into hypoperfused tissue and triggering redox-sensitive signaling.
2. The strongest evidence supports HBOT for reducing necrosis and improving tissue viability.
3. Most adverse effects are predictable and manageable, while serious complications are uncommon.

**Open Questions**

1. What is the optimal “dose–timing” recipe by procedure? (pressure, session length, total number).
2. Which patients benefit most, and how should we select them?
3. Do early biologic effects translate into durable, patient-centered outcomes?
4. Is perioperative HBOT cost-effective in modern pathways?

## Additional files:

***Additional Table 1:*** *Mechanistic evidence of HBOT.*

***Additional Table 2:*** *Randomized controlled trial evidence of HBOT.*

***Additional Table 3:*** *Non-RCT evidence of HBOT.*

## Data Availability

*All data generated or analyzed in this study are included in this published article and its additional files.*
